# Health and Well-Being through COVID-19 Vaccination: Physical, Oral, and Psychological Effects

**DOI:** 10.3390/ijerph20043117

**Published:** 2023-02-10

**Authors:** Kelvin I. Afrashtehfar, Carlos A. Jurado, Salem H. Abu-Fanas, Mohamed A. Jaber

**Affiliations:** 1 Clinical Sciences Department, Ajman College of Dentistry, Ajman University, Ajman City P.O. Box 346, United Arab Emirates; 2 Department of Reconstructive Dentistry and Gerodontology, School of Dental Medicine, University of Bern, 3010 Bern, Switzerland; 3 Department of Prosthodontics, The University of Iowa College of Dentistry and Dental Clinics, Iowa City, IA 52242, USA

## 1. Introduction

The Coronavirus Disease 2019 (COVID-19) pandemic and its evolving variants have spurred a worldwide effort to control its transmission and reduce its impact [1,2,3]. At the forefront of this effort is the administration of vaccines, which have been proven to be safe and highly effective in preventing severe outcomes from the virus [4,5]. This editorial offers valuable insights into the physical, oral, and psychological adverse reactions associated with COVID-19 vaccines, and delves into their mechanisms of their action. Additionally, the manuscript examines the impact of emerging variants on vaccine efficacy and the ethical and legal considerations surrounding their use. The authors also emphasize the ongoing need for monitoring and research to keep pace with the dynamic landscape of the pandemic and the efficacy of COVID-19 vaccines.

## 2. COVID-19 Immunization: Physical, Oral, and Psychological Adverse Reactions

Like any medical intervention, the administration of COVID-19 vaccines can elicit a range of physical, oral, and psychological adverse reactions. Physical adverse reactions may include erythema, tenderness, swelling, pain, fever, chills, headache, myalgia, arthralgia, fatigue, nausea, vomiting, and lymphadenopathy at the injection site [6,7]. Oral adverse reactions can encompass xerostomia, thirst, and alterations in gustation and olfaction [8,9]. Psychological adverse reactions can include vaccine hesitancy [10,11], and anxiety and fear related to the vaccine and its potential adverse reactions [12,13]. However, it is essential to note that most adverse reactions reported have been mild and temporary, and the long-term effects of COVID-19 vaccination remain unknown.

While it is normal to experience some degree of discomfort after receiving a vaccine, healthcare providers should be vigilant in monitoring and reporting any severe or unusual reactions to the appropriate authorities. Individuals who experience symptoms such as headache, abdominal pain, leg swelling, or dyspnea within three weeks of vaccination are generally advised to seek immediate medical attention [14].

Individuals must have accurate and reliable information about the benefits and risks of COVID-19 vaccination to make informed decisions about their health and well-being [15]. Healthcare providers play a pivotal role in educating individuals about the safety and efficacy of COVID-19 vaccines and addressing any concerns they may have. The ongoing monitoring and research of COVID-19 vaccines will continue to provide important insights into their safety and effectiveness, helping to mitigate potential adverse reactions associated with them.

## 3. COVID-19 Prevention Vaccination: Rare Adverse Events and Causality

Serious adverse events, including thromboembolic events, stroke, myocardial infarction, and sudden death, have been reported in rare cases following COVID-19 vaccination [16,17]. Although the incidence of such events remains low, the underlying causality is unclear. Most thromboembolic events have been reported in women under the age of 60, leading researchers to consider a potential connection to the vaccine [18]. However, a definitive causal relationship between these events and COVID-19 vaccination has yet to be established through further investigation and study. It is essential to be aware that some individuals may also experience alterations in their menstrual cycle due to the vaccine [19,20], which could impact hormonal levels.

## 4. Anti-COVID Vaccination: Mechanisms of Action

The COVID-19 vaccines work by triggering an immune response against the Severe Acute Respiratory Syndrome Coronavirus 2 (SARS-CoV-2) virus [21,22]. The vaccines can either deliver viral components, such as viral proteins, or genetic material delivered by a vector, such as a benign virus. These mechanisms allow the immune system to recognize and respond to the virus in the case of future exposure. This process is critical in building immunity against the virus, thus reducing the risk of severe illness and death.

As the SARS-CoV-2 virus continues to evolve, it is essential to understand how this may impact the efficacy of current vaccines. In some instances, booster shots may be necessary to maintain protection against new virus variants [23,24]. To stay ahead of the virus, ongoing monitoring and research are essential in assessing the impact of new variants on vaccine efficacy, while also ensuring the continued safety of COVID-19 vaccines.

## 5. Coronavirus Vaccination: Ethical and Legal Considerations

The rollout of COVID-19 vaccines under experimental use authorization has sparked important ethical and legal considerations related to health and life insurance coverage [25,26]. There is a potential risk that individuals may not be covered for certain conditions if an experimental vaccine is determined to be a contributing factor. This raises concerns for many individuals and highlights the need for clear and transparent communication between vaccine recipients, healthcare providers, and insurance companies. Individuals must take the time to carefully review their insurance policies prior to receiving the vaccine and to have open discussions with their healthcare providers and insurance companies about any concerns they may have [27]. By doing so, individuals can better understand their rights and responsibilities and make informed decisions about their health and well-being.

## 6. Take Home Message

In conclusion, COVID-19 vaccination is a critical component in the fight against the pandemic and is crucial for maintaining overall public health and safety. Despite the potential physical, oral, and psychological adverse reactions associated with the COVID-19 vaccines, as well as the impact of emerging variants, and the unknown long-term effects, the benefits of vaccination are vast. Most importantly, thus far, these benefits have been demonstrated to outweigh any perceived risks. Healthcare providers and public health authorities must work together to ensure that the administration of COVID-19 vaccines is safe and effective while providing accurate information and support to individuals as they make informed decisions about their health and well-being. A collaborative effort between healthcare providers, government bodies, and the public is essential in ensuring widespread vaccine coverage, and ultimately a path toward herd immunity and the end of the pandemic.
